# Rare occurrence of sagittal sinus thrombosis and haemorrhagic infarction with dengue fever: jumping from traditional symptoms to lethal neurological consequences

**DOI:** 10.1097/MS9.0000000000002406

**Published:** 2024-07-23

**Authors:** Khabab Abbasher Hussien Mohamed Ahmed, AlHussein Abbasher, Amira Siddig, Mohammed Abbasher, Abubaker Alsedig Abbasher, Ali Abdelhaleem Omar Ahmed, Zinab Shihab Hamednalla Abdelgader, Esraa Hassan Salih Elhaj, Areeba Ahsan, Ghassan E. Mustafa Ahmed, Abbasher Hussien

**Affiliations:** a Faculty of Medicine, University of Khartoum; b AlYarmouk College; c Faculty of Medicine, AlNeelain University; d Faculty of Medicine, National Ribat University, Khartoum, Sudan; e Faculty of Medicine, Beni-Suef University, Beni-Suef, Egypt; f Foundation University School of Health Sciences, Islamabad, Pakistan

**Keywords:** dengue fever, haemorrhagic fever, ischaemic stroke, sagittal sinus thrombosis, stroke

## Abstract

**Introduction and importance:**

Dengue virus (DENV) is an RNA virus transmitted by Aides mosquito causing dengue fever. There is growing recognition of neurological symptoms associated with DENV infection, some of which might be lethal if left untreated. Case reports describing sagittal sinus thrombosis, as a serious neurologic consequence of dengue infection, are rare. It is still unknown how often sagittal sinus thrombosis occurs and what variables increase the risk in dengue patients.

**Case presentation:**

Herein the authors presented an elderly Sudanese patient diagnosed with dengue fever. He was admitted, then 2 days after admission, the condition was complicated by atrial fibrillation, sagittal sinus thrombosis complicated by massive left temporal lobe infarction with haemorrhagic transformation and recurrent episodes of status epilepticus. After receiving the necessary care, his condition remained the same and no progress or deterioration was seen.

**Clinical discussion:**

Sagittal sinus thrombosis can happen due to several underlying causes. DENV can very rarely lead to such condition. The authors’ patient developed this condition, which was later complicated by ischaemic stroke with haemorrhagic transformation and status epilepticus. In addition to a familial history of DVT and a history of myocardial infarction, our patient also acquired cardiac mural thrombus and DVT throughout his illness, which increased the suspicion of a protein C, protein S, or antithrombin 3 deficiency.

**Conclusion:**

Sagittal sinus thrombosis with haemorrhagic infarction associated with thrombocytopenia is a very rare kind of stroke that occurs in dengue. Dengue as a pathogenic mechanism of ischaemic stroke requires validation with further data.

## Introduction

Highlights

- A rising number of neurological symptoms linked to dengue virus (DENV) infection are being identified; some of these symptoms might be fatal if addressed.
- Sagittal sinus thrombosis, a dangerous neurologic side effect of dengue infection, is very seldom reported in case studies.
- A patient from Sudan who is elderly and has been diagnosed with dengue fever is seen here. Two days after being hospitalised, the patient experienced recurrent bouts of status epilepticus, a large left temporal lobe infarction with haemorrhagic change, and complex sagittal sinus thrombosis.

After malaria, dengue is the second most frequent disease spread by mosquitoes that affects people. Dengue fever viruses; DENV1 to DENV4 are the four closely related but antigenically distinct virus serotypes. Clinical symptoms can vary from a severe haemorrhagic fever to an asymptomatic condition and are carried by Aedes mosquitoes^1^. The WHO revised its categorisation and dengue recommendations in 2009, adding central nervous system (CNS) involvement to the list of symptoms considered severe. There has been a recent shift in the range of clinical signs, with a greater recognition of neurological abnormalities. Neurological manifestations of dengue infection include encephalitis, meningitis, vascular events either haemorrhage or thrombosis, Guillain barre syndrome, and neuro-ophthalmic events, with encephalopathy and encephalitis, being the most frequent neurological side effects of dengue, having a frequency of between 0.5 and 6.2%, according to estimates^2–5^.

Multiple nations across every continent have reported cases of dengue virus (DENV) neurological symptoms. Those as young as three months old and as old as 60 years old are among the affected subjects^4–6^. The following conditions stand alone as risk factors for the neurological consequences of DENV infection: high haematocrit, thrombocytopenia, skin rash, and liver failure^6^. Guillain–Barré syndrome (GBS), myositis, encephalitis, and myelitis are among the several neurological symptoms that have been documented. Infections with DENV2 and DENV3 are mostly linked to such symptoms. Sentinel infections, meningitis, and myelitis have all been linked to these serotypes^7–9^. Additionally, immune histochemistry has identified DENV4 in brain cells as well as in the cerebrospinal fluid (CSF) of encephalitis patients^10,11^.

Sagittal sinus thrombosis followed by stroke is a rarely reported neurological manifestation of dengue infection.

The work has been reported in line with the SCARE 2023 criteria^12^.

### Case presentation

A 66-year-old Sudanese male, who is not known to be diabetic or hypertensive, with a history of myocardial infarction 10 years ago and a family history of DVT. He was on aspirin and atorvastatin as part of his treatment regimen after MI and has been taking these medications for over a decade. The patient’s history or medical records do not contain documentation indicating prior testing for deep vein thrombosis (DVT). He presented with generalised fatigability, headache and high-grade fever associated with nausea, vomiting and sweating. Also, a generalised skin rash with ecchymosis was noticed. Due to the severity of his symptoms, he was immediately admitted to the hospital. After two days in the hospital, his son noticed that he became drowsy and confused and had right-sided weakness involving upper and lower limbs that reached maximum intensity within two days and became completely paralysed. With exception of deviation of the mouth to the left, there were no symptoms in favour of cranial nerves involvement. On the Next day, he experienced recurrent attacks of generalised tonic-clonic seizures that lasted for more than 5 minutes and failed to be aborted by diazepam but responded well to phenytoin infusion and hence was diagnosed as status epilepticus. Following that his condition was static.

**Figure 1:**
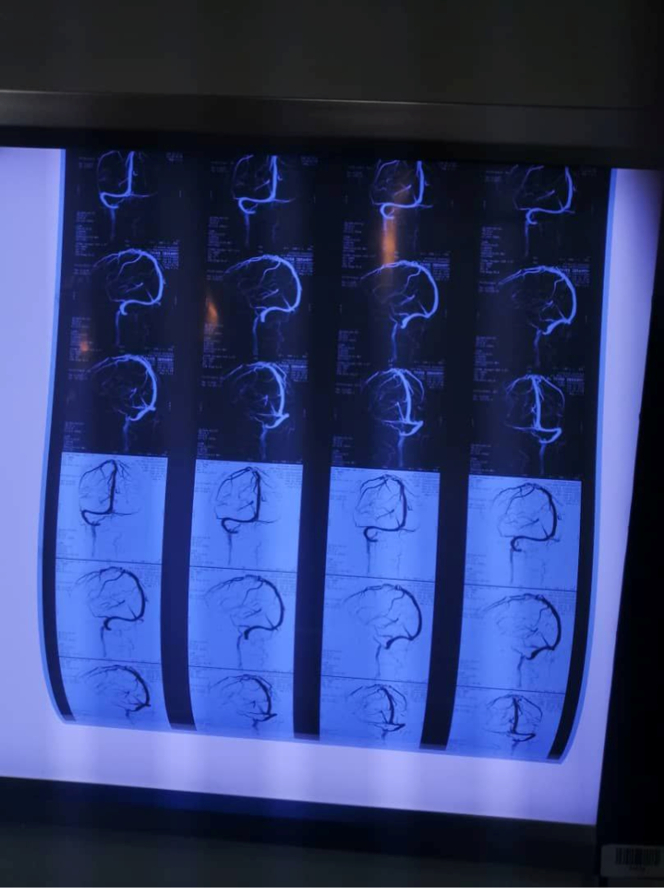
Brain MRV demonstrating sagittal sinus thrombosis (loss of normal signal in the left transverse, sigmoid, and internal jugular veins).

**Figure 2:**
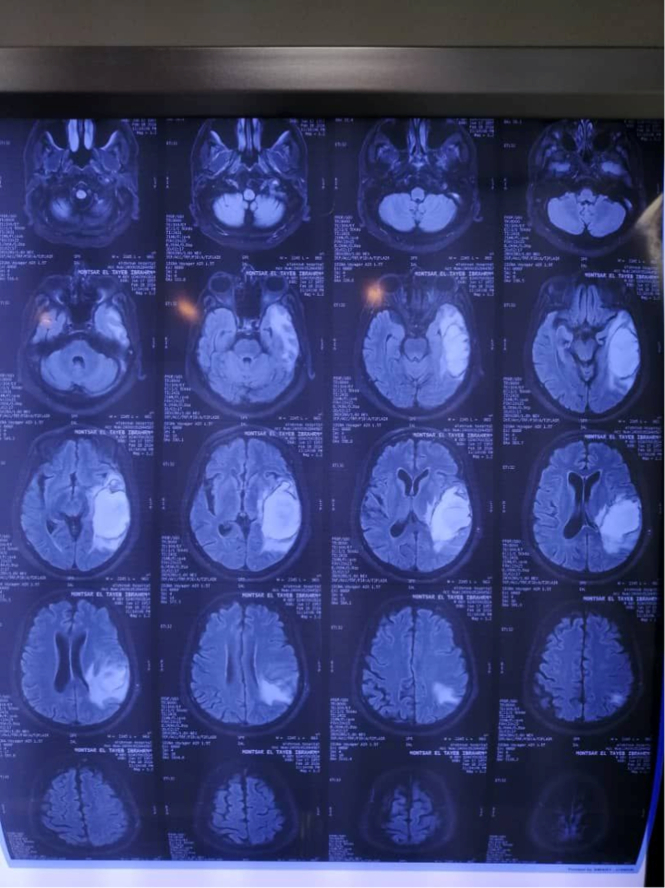
A stunning midline shift and a large finger-like oedema surround a left temporal major infarction with haemorrhagic transformation shown on a brain MRI.

**Figure 3:**
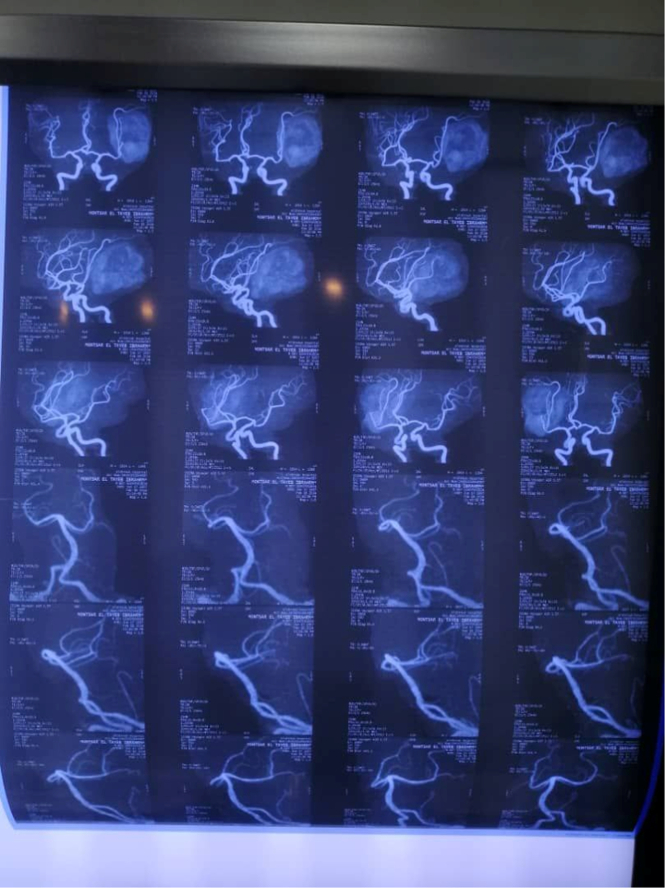
Normal MRA examination of the brain.

Clinical examination on admission revealed an ill febrile confused patient, not pale, jaundiced or cyanosed. Pulse was 100/min regular and large volume; blood pressure was 110/65 mmHg and truncal examination revealed petechial haemorrhage and ecchymosis more than upper & lower limbs. Cardiovascular examination and electrocardiogram revealed atrial fibrillation, while chest examination and X-ray showed right lobar pneumonia. Abdominal examination revealed a palpable non-tender liver. Upon examination of the central nervous system, the patient was confused, and apathetic, no papilloma or neck stiffness was detected and there was right-sided hemiplegia (power grade zero, hypotonia and areflexia). The following investigations were done: Urine general which showed proteinuria, CBC showed leukopenia (3200 per microliter of blood) and thrombocytopenia (80 000 per microliter of blood). Serum albumin was (2.2 g/dl), while AST was high (240 IU/l) and PTT and INR were prolonged. Dengue-virus-specific IgM was positive. Brain MRV showed evidence of sagittal sinus thrombosis (Fig. 1) and brain MRI showed a left temporal massive infarction with haemorrhagic transformation surrounded by gross finger-like oedema with remarkable midline shift (Fig. 2). The presence of sagittal sinus thrombosis, as indicated by the MRV findings, could explain the thrombotic event that affected the venous sinuses in the brain. This condition can cause various neurological symptoms, including headaches. However, there was no specific mention of a history of such headaches prior to the patient’s current Dengue fever infection. Brain MRA was done and found to be normal (Fig. 3). Echocardiography showed evidence of mural thrombus. The leukopenia, thrombocytopenia, hypoalbuminemia, elevated AST, and prolonged PTT and INR are suggestive of systemic inflammation and coagulopathy, commonly observed in severe Dengue fever. In addition, the diagnosis of Dengue fever was confirmed by the presence of Dengue-virus-specific IgM antibodies. The IgM seroconversion in paired serum samples is considered a reliable diagnostic criterion for Dengue virus infection. The positive result supports clinical suspicion and emphasises the significance of Dengue virus as the underlying cause of the patient’s condition.

Blood tests ruled out common differentials such as DIC and other infectious aetiologies. A multidisciplinary approach was employed, involving various medical specialties, to ensure a thorough evaluation of the patient’s condition.

**Figure 4:**
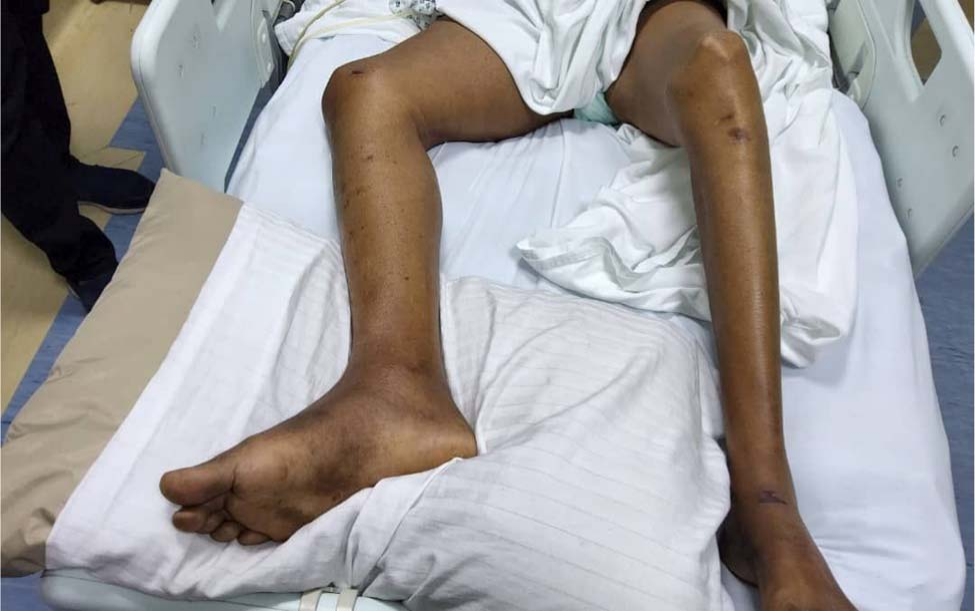
As the patient was admitted to the hospital, their health worsened and they acquired deep vein thrombosis in their right lower limb.

During hospitalisation, the condition became worse and he developed right lower limb DVT (Fig. 4). Glasgow comma scale deteriorated from 11 to 6. In response to the patient’s declining GCS, respiratory support was provided through oxygen therapy delivered via a face mask for adequate oxygenation. Additionally, the physicians closely monitored the patient’s oxygen saturation levels and respiratory status to make necessary interventions, if required. He received intravenous fluids and a nasogastric tube was inserted. He received broad-spectrum antibiotics (Meropenem), dexamethasone and clexane followed by an oral anticoagulant. For the convulsions, Regarding the convulsion diazepam IV was administered and when no response was seen Phenytoin was added, and then he was shifted to levetiracetam. The condition was then static and no deterioration nor improvement was noticed. The patient received appropriate medical care, including oxygen therapy, intravenous fluids, broad-spectrum antibiotics, anticoagulation therapy, and antiepileptic medications. However, the patient’s neurological status did not improve. The medical team carefully monitored the patient’s vital signs, neurological function, and laboratory results but no positive changes were observed. Fortunately, no adverse events or unexpected developments occurred related to the treatment during the hospitalisation period.

## Discussion and literature review

### Overview

Dengue fever, (also known as break-bone fever), is a viral disease transmitted from mosquitoes to humans. Tropical and subtropical regions are where it is more prevalent. Most dengue patients have no symptoms at all. However, the most typical signs and symptoms for those who do include rash, high fever, headache, body pains, and nausea. In one to two weeks, the majority will likewise recover. Some people get severe dengue and require medical treatment. Dengue may be deadly in extreme circumstances^13^.

Dengue can be caused by any of the four serotypes, which are DENV1 through DENV4, as previously mentioned. Dengue fever is an illness brought on by one of them. Infection with the same subtype confers lifelong protective immunity; however, infections from different serotypes are unaffected^14^. When viruses of various serotypes are later encountered, the likelihood of getting dengue haemorrhagic fever is increased if there is a history of prior infection with a different serotype. The phenomenon that is both intriguing and counterintuitive is called “antibody-dependent enhancement” (ADE)^15^, in which mononuclear phagocytes infected with DENV are more effectively infected when heterotypic non-neutralising antibodies bind with them. The outcome worsens clinical symptoms as more host cells get infected and promote viral multiplication. Developing neurological and other issues might result from all of these^16,17^. In light of the ongoing SARS-CoV-2 pandemic, a distinct kind of issue may be anticipated to arise in connection with ADE^18,19^.

There are three classifications for dengue infection according to the 2009 WHO classification: severe dengue, illness with warning signals, and dengue with no warning signs. CNS involvement is now recognised as a symptom of severe dengue in this updated categorisation^1^. When dengue fever infections include many organ systems, the symptoms are referred to as extended dengue syndrome (EDS)^20^. EDS is more likely to occur in certain high-risk populations, including children, the elderly, hemoglobinopathies patients, pregnant women, and those with weakened immune systems^21,22^.

### Neurological complications

The neurological consequences of DENV infection were previously divided into three groups according to their pathogenesis^1,17,18^. These groups included those brought on by metabolic disturbances, such as encephalopathy; those brought on by viral invasions, such as encephalitis, meningitis, myositis, and myelitis; and^3^ those brought on by autoimmune reactions, such as acute disseminated encephalomyelitis (ADEM), optic neuritis, myelitis, and Guillain–Barré syndrome (GBS). Three categories of neurological involvement have been documented more recently by Solbrig and Perng: those involving the CNS and eyes, those connected to peripheral nervous system (PNS) syndromes, and those that happen during the convalescent phase or post-dengue immune-mediated syndromes^19^.

Any one of the three conditions—dengue fever, dengue haemorrhagic fever, and dengue shock syndrome—may affect the central nervous system. According to the WHO, a verified diagnosis of DENV infection is necessary for the diagnosis of any neurological condition linked to the virus. IgM seroconversion in paired serum samples, PCR positive, viral culture positivity, and four times IgG titre rise in paired serum samples are all examples of this. Any one of the following symptoms might indicate dengue’s involvement in the CNS: stiff neck, focal neurological signs, seizures, or reduced state of consciousness (Blantyre coma score < 4 for children under 5 years old, and Glasgow coma score < 14 for children over 5 years old)^1^.

### Pathophysiology

During dengue infection, multiple mediators such as cytokines, chemokines and complement are released, which have vasoactive or procoagulant effects leading to thrombocytopenia, disseminated intravascular coagulation and vasculitis, which may result in stroke^23^. Sagittal sinus thrombosis in dengue infection is thought to be a consequence of severe dehydration resulting from plasma leakage. Several studies reported dehydration-related thrombosis secondary to diabetic ketoacidosis^24^, hyperosmolar nonketotic state, and acute gastroenteritis^25^ has been reported previously. Though the clinical presentation of dengue fever varies from asymptomatic to organ failure, proper hydration is very important in the early stages to prevent such a neurological complication like CVT.

### Case analysis

Our patient presented with fever, joint pain, proteinuria, hypoalbuminemia with remarkable leukopenia and thrombocytopenia and positive Dengue virus IgM. Cardiovascular examination and electrocardiogram revealed atrial fibrillation. Clinical (cardiac) symptoms are a possible presentation for dengue patients, which increases the risk of mortality and disability. Cardiac dysfunction, myocarditis, pericarditis, irregular heartbeats, and hypotension are among the cardiovascular consequences of dengue^16^. It is unknown what causes the heart illness associated with dengue infection. Its aetiology, which causes cardiac damage and consequent conduction anomalies, is influenced by several circumstances. Cardiac involvement can result from direct viral invasion of the heart muscles, cytokine-induced immunological damage, or both^26^.

Our patient later developed sagittal sinus thrombosis with haemorrhagic infarction and post-stroke epilepsy. Rarely does dengue illness led to stroke. The most frequent kind of stroke that is seen is haemorrhagic stroke^27^. The opposite of a sinus thrombosis and ischaemic stroke is thought to be uncommon^27^. This could, however, be the result of the fact that DENV screening is not typically performed on ischaemic stroke patients. Other authors have reported that neurological symptoms can appear at any stage of dengue infection or after the general symptoms of the virus. Ischaemic stroke during dengue infection is an acute neurological manifestation^27^. According to previous research^27^, patients who have a history of comorbidities, are male, older than 60, have severe dengue or dengue haemorrhagic fever, and are at a higher risk of experiencing an ischaemic stroke due to DENV infection. Our patient falls into this category.

On the other hand, the serotypes of DENV3 and DENV2, and to a lesser degree, DENV4 and DENV1, have been linked to neurological symptoms. It is still unclear, nevertheless, why ischaemic stroke is more commonly linked to the DENV serotype^27^. It’s unclear what exactly causes the ischaemic stroke linked to dengue. The causes postulated include a transitory hypercoagulable condition after plasma leakage and immune-mediated arteritis^28^. The suggestion that corticosteroids can alleviate vasculitis and clinical symptoms is supported by the previously reported immune-mediated response^29^.

According to reports, NS1 causes coagulation abnormalities linked to dengue through some mechanisms, including cytokine storm caused by complement and macrophage activation, which increases permeability and endothelial damage^30^. These processes result in plasma leakage and bleeding that are typical of dengue infection, which might account for the greater frequency of haemorrhagic stroke described in comparison to ischaemic stroke linked to DENV^31^. Sinus thrombosis commonly occurs in dehydrated patients and can be due to ear, nose, gums or eye infections. Also, sinus thrombosis can occur during pregnancy or due to the use of contraceptive pills. Rarely HIV, Brucellosis and sickle cell anaemia can cause sinus thrombosis^32^. Additional elements that contribute to procoagulant haemostasis in dengue infection include the production of thrombomodulin by infected endothelial cells and the loss of endothelial non-thrombotic factors, this can lead to the consumption of protein C, protein S and antithrombin 3 and lead to the formation of sinus thrombosis^31^. Our patient had a history of myocardial infarction and a family history of DVT and during the course of the disease, he developed DVT and cardiac mural thrombus raising the possibility of protein C, protein S or antithrombin 3 deficiency.

After receiving treatment in accordance with WHO standards, our patient’s condition stabilised rather than worsened. The final diagnosis was dengue infection-related sagittal sinus thrombosis. This rare presentation of dengue fever complicated with sagittal sinus thrombosis is compatible with previous literature. A previous study reported an unusual presentation of a 16-year-old boy with Dengue fever who developed bilateral papilledema, left eye restricted abduction, and his MRI brain with venogram showed bilateral transverse sinus thrombosis. He was diagnosed as cerebral venous thrombosis due to dehydration with underlying dengue infection^33^.

## Conclusion and recommendations

It is yet unclear how DENV infection affects the neuro-pathogenesis. The development of dengue-related neurological disorders is likely influenced by both host and viral factors. There might be three possible processes at work: the virus’s direct invasion of the central nervous system, immunological responses, and metabolic changes. Even though DENV has always been regarded as non-neurotropic, neurological involvement, the presence of CSF viral particles, and BBB degradation all appear to point to direct viral neurotropism. Neurologists should think of this complication in any patient with Dengue fever who shows deterioration of level of consciousness.

## Ethical approval

Ethical approval was not needed as this is a case report.

## Patient consent

Written informed consent was obtained from the patient for publication and any accompanying images. A copy of the written consent is available for review by the Editor-in-Chief of this journal on request.

## Source of funding

The study was funded by the authors themselves.

## Author contribution

K.A.H.M.A., A.A., A.S., and M.A. wrote the first draft, visualised, validated, conceptualised, did the investigations, and administered the project, while A.A.A., A.A.O.A., Z.S.H.A., E.H.S.E., G.E.M.A. and A.A. wrote the first draft, visualised, validated, conceptualised, and critically reviewed, and A.H. Wrote the first draft, validated, visualised, conceptualised, and supervised the study. All authors participated in the examination, investigation, management and writing of this case.

## Conflicts of interest disclosure

The authors declares no conflicts of interest.

## Research registration unique identifying number (UIN)

1. Name of the registry: NA.
2. Unique Identifying number or registration ID: NA.
3. Hyperlink to your specific registration (must be publicly accessible and will be checked).

## Guarantor

Khabab Abbasher Hussien Mohamed Ahmed.

## Data availability statement

The dataset used and/or analysed during the study is available from the corresponding author upon reasonable request.

## Provenance and peer review

Not commissioned, externally peer-reviewed.
